# The Dependence of the Properties of Recycled PET Electrospun Mats on the Origin of the Material Used for Their Fabrication

**DOI:** 10.3390/polym14142881

**Published:** 2022-07-16

**Authors:** Ewa Kijeńska-Gawrońska, Katarzyna Wiercińska, Monika Bil

**Affiliations:** 1 Centre for Advanced Materials and Technologies CEZAMAT, Warsaw University of Technology, Poleczki 19, 02-822 Warsaw, Poland; monika.bil@pw.edu.pl; 2 Faculty of Materials Science and Engineering, Warsaw University of Technology, Woloska 141, 02-507 Warsaw, Poland; katarzynka.wiercinska@gmail.com

**Keywords:** polyethylene terephthalate (PET), recycling, electrospinning, fibers

## Abstract

Plastic materials are one of the significant components of construction materials omnipresent in all areas of the industry and everyday life. One of these plastics is polyethylene terephthalate (PET). Due to its processing properties, with a simultaneous low production cost, PET has been used in many industrial applications, including the production of various types of bottles. Moreover, the high consumption of PET bottles causes the accumulation of large amounts of their waste and necessitates finding an effective way to recycle them. Electrospinning is a well-known non-complicated method for the fabrication of nonwovens from polymers and composites, which can be utilized in many fields due to their outstanding properties. In addition, it might be a promising technique for the recycling of plastic materials. Therefore, in this study, the electrospinning approach for the recycling of two types of PET bottle wastes—bottles made of virgin PET and bottles made of recycled PET (PET bottles) has been utilized, and a comparison of the properties of the obtained materials have been performed. The fibers with diameters of 1.62 ± 0.22, 1.64 ± 0.18, and 1.89 ± 0.19 have been produced from solutions made of virgin PET granulate, PET bottles, and PET bottles made of recycled bottles, respectively. Obtained fibers underwent morphological observation using a scanning electron microscope. Physico-chemical properties using FTIR, gel chromatography, and differential scanning calorimetry have been evaluated, and mechanical properties of obtained mats have been investigated. Cytotoxicity tests using the L929 mouse fibroblast cell line revealed no cytotoxicity for all tested materials.

## 1. Introduction

Polymer materials, sometimes called the *material of the millennium* [1], are lightweight, pliable, durable materials, easily convertible to many various forms with a low cost of production. Therefore, a broad spectrum of applications was found in industry and everyday life. Their invention and progress in processing have revolutionized the modern world and pushed the limits of human capabilities, as wood, ceramics, glass, copper, bronze, iron or steel did in earlier stages of our civilization [2]. Modern science has not yet found substitutes that can replace plastics in terms of their multi-functionality, features, and properties. However, the main drawback of their utilization is the accumulation of plastic waste in the environment and, of course, the human factor in its improper storage, processing, and littering. The production of the polymers worldwide reached 359 MTs in 2018, with an estimated 18% involvement of polyethylene terephthalate (PET). Only a small amount of its waste, mainly bottles, has been reported to be recycled [3]. Therefore, many actions have been undertaken to reduce its waste and develop alternative recycling methods for PET waste reduction.

PET material used for bottle production is a semi-crystalline thermoplastic with good mechanical properties and is highly resistant to environmental conditions and many chemical reagents. Moreover, it can be recycled multiple times, and recently plastic bottles can be recycled into other bottles, sleeping bags, t-shirts, clothes insulation, etc. [4]. Nonwovens obtained by processing PET waste could also be of great potential as a substrate for various kinds of fibers applied for water filtration and treatment and air filtration. Furthermore, it may also find application in hygienic membranes utilized in the cosmetic industry and beautician services or fabrics for respiratory and medical protection. Their porous structure and high tensile strength allow it for many applications, and its main advantage is low cost and easy access to the material (wastes).

Electrospinning is a versatile, scalable (from laboratory to industry) method for fiber fabrications having diameters ranging from nanometers to sub microns, with controlled morphology, orientation and structure features [5]. It is frequently used for the production of nonwoven for biomedical application as tissue engineering scaffolds [6], wound dressings [7], multisource catalysis [8], filtration [9], water treatment [10] or bio-active agent delivery carrier [11]. On the other hand, virgin PET granulate has been utilized to produce wound dressings [12] and blood vessel grafts [13]. Attempts have also been made to utilize recycled PET bottles for the production of filtering membranes for air and water filtration [14,15]. The mentioned studies have drawn attention to the importance of using post-consumer PET bottles to create new materials, which seems to be the right course of action from the circular economy perspective. These researches focus mainly on the utilization of bottles made of virgin PET (which might be correlated with the inexistence of bottles made out of 100% recycled PET by the time that the studies have been conducted), and comparison to pristine material has not been performed. Moreover, most of the studies regarding the production of fibrous materials from recycled PET use trifluoroacetic acid (TFA) or a mixture of dichloromethane with trifluoroacetic acid for electrospinning of the fibers [16]. When using the PET alone, the choice of TFA does not influence material at a high level. However, if the designed material aims to be a composite with metal oxides, the use of TFA is inadvisable. Therefore, in the presented studies, 1,1,1,3,3,3-hexafluoro-2-propanol (HFP) has been employed to prepare the solution for electrospinning. Though its toxicity is known, no remaining HFP stays in resultant fibers, and its industrial application is possible since it is non-flammable. This property is usually crucial for the utilization of solvents in the industry. It is also utilized to prepare a composite solution containing metallic nanoparticles [17].

This study aimed to use the waste PET bottles made of virgin PET granulate and PET bottles made out of recycled bottles to fabricate electrospun nonwovens and to compare their properties with each other, along with a comparison to electrospun mats produced from PET granulate. In this way, the dependence of mats’ properties on the origin of the materials used for their production could be evaluated, and the question of possible recycling of already recycled bottles could be answered. Moreover, by characterizing the main properties of PET mats, we were able to investigate whether the obtained materials might be promising candidates for application for hygienic membranes, air and water filters or other products that stay in contact with human tissues, such as skin, without causing cytotoxic reactions.

To the best of the authors’ knowledge, this is the first time that bottles made entirely of recycled PET bottles have been utilized for fibers fabrication, and detailed studies on the comparison of properties of PET fibers made of PET of different origins have been performed.

## 2. Materials and Methods

### 2.1. Materials

Polyethylene terephthalate in the form of granules for water bottle manufacturing (LighterTM C93- Equipolymers) was purchased from RESINEX Poland Sp. z o.o. (Warszawa, Poland). Recycled bottles utilized in this study were made from virgin PET (granulate) and entirely (in 100%) from recycled PET. Both types were purchased from the same local supplier, and before use, they contained mineral water. In all experiments, granules of PET were used in the received form, while bottles were firstly cleaned with detergent and deionized water, dried at 37 °C, and cut into square flakes of about 5 × 5 mm. Additionally, 1,1,1,3,3,3-hexafluoro-2-propanol (HFP) utilized as a solvent for electrospinning was obtained from abcr GmbH (Karlsruhe, Germany). HPLC-grade chloroform and absolute ethanol were purchased from Chempur (Piekary Slaskie, Poland). Polystyrene standards utilized for molecular weight measurements and phosphate-buffered saline (PBS) were purchased from Sigma-Aldrich (St. Louis, MO, USA). L929 murine fibroblast cells were obtained from American Type Culture Collection (ATCC) (Manassas, VA, USA). RPMI 1640 medium, fetal bovine serum (FBS), penicillin-streptomycin solution (PS), and trypsin-EDTA were bought from Thermo Fisher Scientific (Waltham, MA, USA). CellTiter 96 Aqueous One solution was purchased from Promega (Madison, WI, USA). Sodium chloride 0.9% solutions were obtained from Baxter (Deerfield, IL, USA).

### 2.2. Characterization of Pristine Unprocessed Materials

#### 2.2.1. Gel Permeation Chromatography (GPC)

Gel permeation chromatograph (Agilent Technologies, Singapore) using a refractive index detector has been utilized to determine the average molecular weights (Mw, Mn) of the PET granulate and PET flakes derived from both types of bottles. First, 4 mg of PET granules were added to 100 µL HFP, heated to 50 °C, and mixed for 30 min to enable dissolution. Then, the solution was mixed with 1.9 mL HPLC-grade chloroform and mixed for another 24 h. In the case of PET bottles, flakes were added to 100 µL HFP and mixed for 30 min, followed by the addition of 1900 µL of HPLC-graded chloroform. Each solution was mixed to obtain the concentration of 2 mg/mL (*w*/*v*). The final step of preparation was filtration through a 0.22 µm porous filter. During testing, 100 µL aliquots of each type of PET solution were inoculated into chloroform and further separated on two linear coupled SEC columns (PLgel 5 mm MIXED-C, Agilent Technologies, Cheadle, UK, 300 × 7.5 mm) at 35 °C and a flow rate of about 0.7 mL/min. Calibration of the system was performed using three polystyrene standards with known molecular weights (Mp ranging from 500 to 1,800,000 g/mol).

#### 2.2.2. Differential Scanning Calorimetry (DSC)

The crystallinity of the pristine materials before processing was evaluated using a differential scanning calorimeter Q2000 (TA Instruments, New Castle, DE, USA) under a nitrogen flow of 50 mL/min. Specimens with the weight of 6–8 mg were heated in aluminum pans from 30 to 300 °C with a rate of 10 °C/min, then cooled back to 30 °C and heated back to 300 °C. Collected data from the second heating run were analyzed using software provided by the equipment manufacturer (TA Universal Analysis).

#### 2.2.3. Surface Hydrophilicity

The surface hydrophilicity of three types of unprocessed materials was assessed through water contact angle (WCA) measurements, and WCA was measured at RT by a sessile drop method using the OCA 20a goniometer (DataPhysics, Filderstadt, Germany). The water droplet size was equal to 1 µL with a dosing rate of 1 µL/s. Images of the droplet shape in contact with material were taken using a CCD camera running in real-time and saved for further analysis.

### 2.3. Optimization of PET Mats Electrospinning

To evaluate the optimal concentration of virgin PET granulate and flakes made out of two types of bottles to obtain uniform fibers, the optimization of the paramaters of the electrospinning process with the usage of different concentrations of the solutions was performed. The summarized list of the utilized solution and parameters has been presented in Table 1. For the preparation of the virgin PET granulate solution first pellets were mixed with 1,1,1,3,3,-hexafluoro-2-propanol at concentrations of 10, 15, and 20% (*w*/*v*) and stirred for 30 min at 50 °C. Furthermore, the solution was stirred for another 24 h at room temperature. In the case of both types of bottle flakes, the desired amount of flakes has been added to HFP to obtain concentrations of 10, 15, and 20% and stirred at RT for 24 h without previous heating. Electrospinning of all types of PET was conducted under 12 kV, with a 1.0 mL/h flow rate using a 27 G flattened needle to a collector distance of 13 cm. A steel plate covered with aluminum foil has been used for collecting the fibers during all the experiments. All processes were carried out under environmental conditions with 23–33% humidity and a temperature of 22–24 °C.

After electrospinning, all samples were evaluated under the scanning electron microscope (SEM, PhenomX, Eindhoven, The Netherlands) after sputter coating with a 14 nm layer of gold (sputter coater Leica EM SCD 500, Leica Mikrosysteme GmbH, Wien, Austria), at an accelerating voltage of 10 kV. Analysis of images allows for the selection of optimal concentration for the PET mats preparation for further studies. For the samples revealing uniform morphology, fibers’ diameters were examined from the obtained SEM images using ImageJ software (National Institute of Health, Bethesda, MD, USA). The average diameter was measured based on the calculation of 100 randomly selected fibers, and the distribution histograms were prepared.

### 2.4. Characterization of Fabricated Mats

#### 2.4.1. Gel Permeation Chromatography (GPC)

Gel permeation chromatography was performed as described for pristine materials and 4 mg of each type of fabricated mats, namely gPET, PET, and rPET, were used.

#### 2.4.2. Differential Scanning Calorimetry (DSC)

The crystallinity of the PET mats, similarly to granules and flakes, was evaluated during annealing using a differential scanning calorimeter.

#### 2.4.3. Fourier Transform Infrared (FTIR) Spectroscopy

Fourier transform infrared spectrophotometer (Thermo Fisher Scientific model Nicolet 6700) was utilized to collect infrared spectra of fabricated electrospun gPET, PET, and rPET mats. Measurements were carried out using the attenuated total reflectance (ATR) mode, and each sample was scanned 64 times at a resolution of 4 cm^−1^ over the wavenumber range of 4000–400 cm^−1^.

#### 2.4.4. Surface Hydrophilicity

The surface hydrophilicity of three types of fabricated mats was measured following the procedure used for the granulate and bottles’ flakes.

#### 2.4.5. Tensile Testing

The tensile properties of the electrospun PET mats were evaluated using a tensile testing machine Instron 5943 (Instron, Norwood, MA, USA). The samples for mechanical testing were cut to 20 mm in length and 4 mm in width and attached to the hydraulic clamps. The tests were performed at a 5 mm/min crosshead speed at RT and ambient humidity.

### 2.5. Cytotoxicity Evaluation

The cytotoxicity of the gPET, PET, and rPET mats was evaluated according to ISO 10993-5 standard [18]. First, L929 mouse fibroblasts were cultured in RPMI 1640 supplemented with heat FBS and 1% PS at 37 °C in a 5% CO_2_ incubator. Fibroblasts were cultivated for 48–72 h for the attainment of sufficient confluence of the cells. After their detachment using 0.05% trypsin-EDTA, cells were seeded in 96-well plates at a concentration of 10^4^ in 100 µL of complete media. At the same time, gPET, PET, and rPET mats were exposed to UV for 30 min on each side and rinsed three times with RPMI 1640 with 1% PS to sterilize the samples. Extracts were derived by soaking the mats separately in a complete medium of RPMI1640, 10% FBS, and 1% PS. Referencing ISO standard guidelines, the mass of specimens within the extraction media was altered to 100 mg/mL (ISO 10993-12 [19]). Furthermore, specimens were incubated for 24 h in an incubator. Obtained 24 h extracts were utilized in dilutions of 1, 2.5, 5, and 10x. After aspiration of cell culture media, 100 µL of each extract or its dissolution was added to cells, and fibroblasts were further incubated for another 24 h. Thereafter, media in each well was replaced with 100 μL of 20% MTS solution in RPMI1640 and incubated for 120 min at 37 °C. Then, absorbance of the aliquots was measured at 490 nm using a microplate reader (Spark, Tecan Austria GmbH, Grodig, Austria). The cytotoxicity results are presented as the percentage of the viability cultured in control condition of complete medium.

### 2.6. Statistical Analysis

Performed experiments were carried out at least in triplicates, and data are expressed as mean ± standard deviation (SD). Post-hoc one-way ANOVA with a Tukey-Kramer pair-wise comparison have been employed for statistical analysis. A value of *p* ≤ 0.05 is considered statistically significant, and additional significance is indicated with ** *p* < 0.01 and *** *p* < 0.001. All statistical analyses were calculated using GraphPad Prism version 9.2.0 for Mac OS X (GraphPad Software, La Jolla, CA, USA).

## 3. Results and Discussion

### 3.1. Properties of Pristine Materials

To make a characterization of all three types of polyethylene terephthalate, namely granulate of virgin PET, PET bottles, and PET bottles made of recycled PET, gel permeation chromatography, differential scanning calorimetry, and contact angle measurements were performed. Obtained data allowed for molecular weight and crystallinity evaluation, and the results are summarized in Table 2. It can be seen that no significant differences have been observed in the case of molecular weight of all three forms of PET. Moreover, both types of bottles exhibited higher crystallinity than granulate virgin PET. This can be attributed to the mechanism of PET bottle manufacturing. The process of bottle production takes two stages: (i) Melting of the granulate at around 280 °C and processing them into preforms, and (ii) and heating the preforms to about 110 °C and blowing them into bottles. This “stretch blow-molding process” causes the partial crystallization of PET, which after that improves its durability, thermal and barrier anti-carbon dioxide, and oxygen properties [20].

Hydrophilicity measurements revealed the hydrophobic nature of the surface of all three types of unprocessed materials.

### 3.2. Optimization of Electrospinning

**Figure 1:**
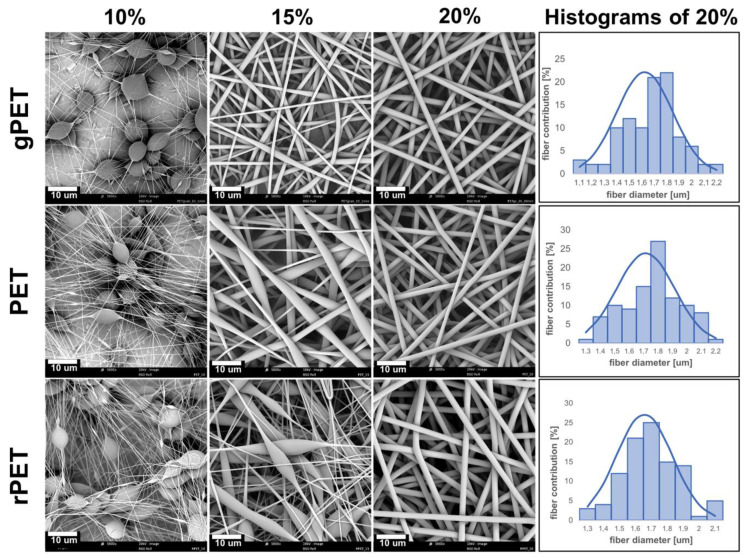
Morphology of obtained fibers during the optimization of electrospinning of PET granulate (gPET), PET bottle flakes (PET), and flakes from bottles made of recycled PET (rPET); histograms obtained for the samples made of 20% PET solutions.

Optimal concentrations for the solutions of virgin PET granulate and both types of PET bottles’ flakes were chosen on the basis of the fiber morphology. Figure 1 presents the morphology of the fibers obtained in the optimization process and the distribution of fiber diameters for 20% solutions. As it can be observed, fibers electrospun from 10% (*w*/*v*) solutions, regardless of the utilized type of PET, consisted of large beads connected with fragile fibers. Increasing the solution concentration to 15% (*w*/*v*) resulted in more uniform fibers, with spindle-like beads formed within their structure. Furthermore, an increase to 20% (*w*/*v*) allowed for the attainment of the homogenous structure of non-beaded fibers. In this way, the optimal parameters have been optimized to produce fibrous mats of uniform fibers, and mats made of 20% solutions were chosen to produce the fibers for further studies. The diameters of the fibers made of virgin PET granulate (gPET), PET bottle flakes (PET), and flakes from bottles made of recycled PET (rPET) were 1.62 ± 0.22 µm, 1.64 ± 0.18 µm, and 1.89 ± 0.19 µm, respectively.

Moreover, the performed Shapiro-Wilk test revealed that obtained distribution of diameters could be described as normal. Based on the properties of normal distribution, it can be concluded that 95% of all diameters in tested mats lie within two standard deviations away from the average. Obtained fibers had larger diameters than fibers obtained by Zander et al. from HFP solutions of PET bottles ranging from 105.5 ± 49 nm to 1039.5 ± 326 nm. However, they used the addition of TBAC salt to decrease bead size and fiber diameters.

### 3.3. Physico-Chemical and Mechanical Properties of Electrospun Fibrous Mats

To investigate the influence of the electrospinning process on the molecular weight of the poly (ethylene terephthalate), gel permeation chromatography was performed for mats obtained from all types of PET, and molecular weight values have been listed in Table 3. Results obtained for gPET show that the electrospinning process caused a 27% decrease in the M_w_, a slight increase of M_n_, and a decrease in PDI, compared to PET granulate, which suggests that more uniform macromolecules were observed in the polymer matrix. This might be attributed to random chain scission of PET macromolecules followed by the dissolution of the part of the degraded material having the shortest chain lengths [21]. The DSC results confirm this thesis as the gPET mat is amorphous, which indicates that easier crystallizable short molecules were removed. As stated in the Materials and Methods section, the preparation of PET granulate solution required heating of the granulate in HFP at 50 °C for 30 min during mixing. Moreover, unprocessed PET granulate exhibited only 12% of crystalline phase, which is considerably less compared to the other two types of utilized PET, which showed around 22% crystallinity, and it has been reported that more amorphic materials tend to degrade faster than their more crystalline counterparts due to their increased susceptibility to solvents [22]. Unprocessed PET bottles of both types exhibited similar molecular weight as 56,600 and 58,200 g/mol for PET bottles and bottles made of recycled PET, respectively, and it remained almost unchanged after electrospinning. Therefore, it might be concluded that the fabrication of the mats did not entail any significant change in the polymers’ macromolecular structure.

**Figure 2:**
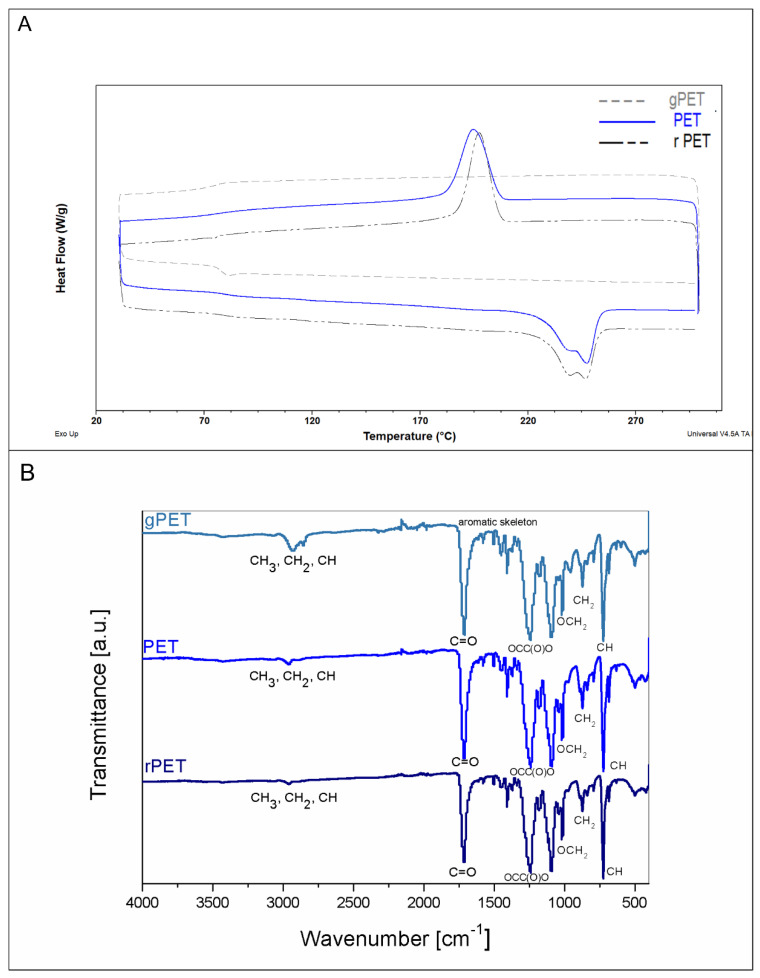
(**A**) Comparison of DSC curves and (**B**) comparison of FTIR spectra for mats made of different types of PET.

Based on DSC curves obtained for all the tested materials, characteristic temperatures, such as glass transition (T_g_), crystallization temperature (T_c_) or melting temperature (T_m_) with melting enthalpy (ΔHm), were determined. Employing melting enthalpy, the crystallinity of the materials was calculated. In Figure 2A, a comparison of DSC curves obtained for different PET forms has been presented, and Table 3 sums the values of characteristic parameters obtained from these curves. The gPET mat possessed a glass transition temperature of 77.2 °C, which is comparable to other samples and unprocessed materials, as well. There are no peaks from melting of the crystalline phase and crystallization, thus the conclusion is that the mat from gPET was amorphous in structure within the range of temperatures tested. This could have two reasons: As the solvent evaporated rapidly in the electrospinning process performed at the room temperature, macromolecules did not have enough time and energy to arrange appropriately to form a crystalline structure, and as the PET granulate is a pure polymer, there were no nucleating agents, that could start the crystallization process [22]. As can be seen on the graph in Figure 2A, a comparison of PET and rPET revealed that there were no noticeable changes in the shape of DSC curves for these materials. Characteristic temperatures and enthalpy confirm that both mats produced from PET bottles possess almost the same glass transition temperature (T_g_ at about 80 °C), crystallization temperature (T_c_ at about 195 °C), melting temperature (T_m_ at about 247 °C), and melting enthalpy, which indicates that their crystallinity is also very similar (22.2% for PET and 22.4% for rPET). Both values are almost the same as those of unprocessed PET and rPET (Table 1).

FTIR spectra were collected and analyzed to determine if there are any differences between gPET, PET, and rPET chemical structures. Characteristic peaks have been marked, and Figure 2B shows a comparison of spectra obtained for gPET, PET, and rPET mats. Obtained data were compared to the literature finding to identify characteristic bands. Band 724 cm^−1^ was assigned to the interaction of polar ester groups with benzene rings, while 793 cm^−1^ was matched with vibrations of two adjacent aromatic hydrogen atoms in *p*-substituted compounds and aromatic bands. Absorption bands 839, 873, and 1016 cm^−1^ are characteristic of aromatic rings. Other peaks significant for material identification are at 1042 and 1093 cm^−1^ for the methylene group and vibrations of ester C-O bond; 1117 and 1182 cm^−1^ for the terephthalate group. Bands 1340, 1409, and 1452 are assigned to a few different phenomena: Stretching of CO group, deformation of OH group, and bending of the ethylene glycol segment of the molecule. Additionally, 1505 and 1578 cm^−1^ are characteristic bands for vibrations of an aromatic skeleton with stretching of C=C bond, 1714 cm^−1^ is assigned to stretching of C=O bond from carboxylic acid group, and the peak around 2950 cm^−1^ is from stretching of the C-H bond [23]. No significant differences were detected between the gPET, PET, and rPET samples.

To investigate the wettability of electrospun mats, water contact angle measurements have been performed, and the obtained values of WCA are listed in Table 3. Obtained data indicate that gPET, PET, and rPET mats exhibited WCA approximately above 130° (with no significant difference), which indicates their hydrophobic behavior. It is also consistent with the literature, where values of 131 and 133° are reported [24,25].

**Figure 3:**
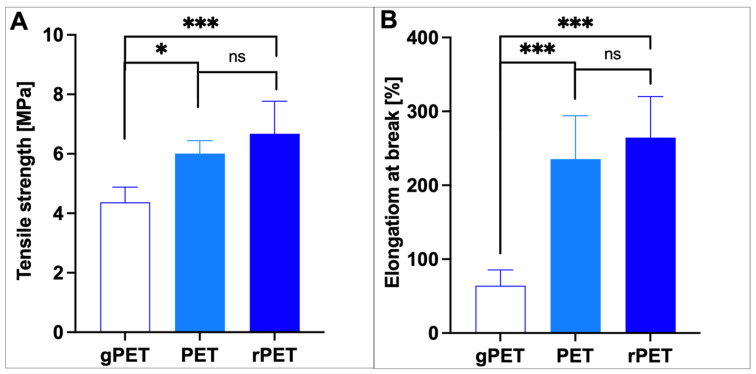
Comparison of (**A**) tensile strength and (**B**) strain at break values for investigated mats. Statistical difference is indicated with * *p* ≤ 0.05, *** *p* < 0.001, “ns” indicates no statistical difference.

Tensile tests have been performed to obtain information about the stiffness and the elasticity of the studied mats. Tensile strength [MPa] and strain at break [%] for all electrospun mats were measured and are compared in Figure 3A,B. Mechanical tests revealed how the form of PET influenced tensile strength and strain at the break of PET. The gPET mat exhibited significantly decreased mechanical properties than the remaining materials (tensile strength at ~4,37 MPa and strain at the break at 64%). It can be assigned to its amorphous structure. It can also be noticed that the rPET showed higher values for both studied parameters than the PET. It might result from chain extenders used in the recycling of PET, as they can also cause branching of the polymer, which can increase mechanical properties [26]. Considering the usefulness of the fabricated materials in applications, such as filters or hygienic membranes, their mechanical properties are of great importance. Values of the mechanical strength obtained for mats made of bottles reached a higher level, with a tensile strength of 6.01 ± 0.43 MPa for PET and 6.67 ± 1.09 for rPET, than the tensile strength reported for electrospun filters made of PAN or PLLA, possessing tensile strength of 3.8 and 2 MPa, respectively [27,28]. Moreover, electrospun mats made of bottles exhibited very high elongation at break of 235 and 264% for PET and rPET, respectively, which was higher than the elongation of PET electrospun membrane filters obtained by Bonfim et al. [14].

### 3.4. Cytotoxicity Evaluation

**Figure 4:**
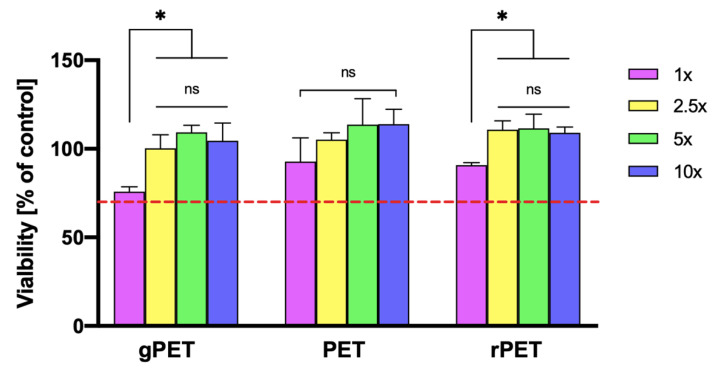
Cytotoxicity (% viability) of L929 fibroblast cell line treated with gPET, PET or rPET mats solute aliquots [1× (undiluted), 2.5, 5, and 10× indicate dilution factor of the solutes]. Red line indicates 70% of the control value. Statistical difference is indicated with * *p* ≤ 0.05 and “ns” indicates no statistical difference.

To evaluate the cytotoxicity of produced materials, in vitro studies using L929 mouse fibroblast cell lines have been performed. ISO 10993-5 standard specifies the reduction of cell viability by more than 30% as exhibiting a cytotoxic effect. Therefore, materials demonstrating the viability of the cells cultured with contact with materials’ extract of less than 70% of the cell control are considered cytotoxic [17]. No extract from gPET, PET, and rPET mats shows the viability of less than 70% (Figure 4). The lowest viability of 76% has been observed for 1x solute extract of gPET. However, from 2.5x onwards, the viability was around 100%. L929 cells cultured with non-diluted extract of PET and rPET exhibited the viability of 93 and 91%, respectively. As a result, it can be concluded that mats prepared from PET granulate, PET from bottles, and PET from bottles made of recycled PET can be considered non-cytotoxic and might find the application in hygienic products of short intended contact time (i.e., surgical masks [14]).

## 4. Conclusions

In the presented study, electrospun fibers made of PET of different origins have been produced and characterized to investigate the impact of PET substrate on the obtained fibrous mat. Performed optimization of the electrospinning process allowed for the selection of optimal parameters for all three studied types of poly (ethylene terephthalate): PET granulate, PET bottles, and bottles for recycled PET. Physico-chemical characterization and mechanical studies show superior properties of PET and rPET mats compared to mats made of PET granulate. Moreover, cytotoxicity evaluation has shown their non-toxicity, confirming their potential use for materials that can be in contact with human tissue, such as skin with limited contact devices, such as masks.

Future studies on the potential utilization of the fibers made of PET from the bottles should focus on the development of antimicrobial and filtration layered fabrics coupled with particles (i.e., metal particles) or other bio-active agents (i.e., enzymes), which could find application for fabrication of membranes for respiratory protection, air, and water filtration or hygienic materials.

## Figures and Tables

**Table 1:** Parameters for three types of PET optimization.

	PET Granulate			PET Bottles			Bottles from Recycled PET		
*Concentration* [% (*w*/*v*)]	10	15	20	10	15	20	10	15	20
*Voltage* [kV]	12	12	12	12	12	12	12	12	12
*Flow rate* [mL/h]	1	1	1	1	1	1	1	1	1
*Needle diameter* [G]	27	27	27	27	27	27	27	27	27
*Needle to* *collector distance* [cm]	13	13	13	13	13	13	13	13	13

**Table 2:** Properties of unprocessed virgin PET granulate, PET bottles, and PET bottles made of recycled PET.

*Properties*	PET Granulate	PET Bottles	Bottles from Recycled PET
*M_w_* [g/mol]	61,800	56,600	58,200
*M_n_* [g/mol]	16,300	17,400	13,400
*PDI*	3.784	3.250	4.091
*Crystallinity* [%]	12	22	21.6
*T_g_* [°C]	79.74	82.88	82.23
*T_m_* [°C]	246.91	247.10	246.68
*Water contact angle* [°]	69 ± 7	81 ± 3	79 ± 7

**Table 3:** Properties of gPET, PET, and rPET mats.

*Properties*	gPET	PET	rPET
*M_w_* [g/mol]	49,000	57,300	57,900
*M_n_* [g/mol]	17,900	21,000	13,100
*PDI*	2.747	2.718	4.415
*Crystallinity* [%]	-	22.2	22.4
*T_g_* [°C]	77.17	80.36	79.03
*T_c_* [°C]	-	195.6	196.18
*T_m_* [°C]	-	247.62	246.7
Δ*H_m_* [J/g]	-	31.1	31.39
*Water contact angle* [°]	138 ± 6	131 ± 3	132 ± 7

## Data Availability

The data presented in this study are available on request from the corresponding author.
